# *De Novo* Transcriptome Sequencing of Desert Herbaceous *Achnatherum splendens* (Achnatherum) Seedlings and Identification of Salt Tolerance Genes

**DOI:** 10.3390/genes7040012

**Published:** 2016-03-23

**Authors:** Jiangtao Liu, Yuelong Zhou, Changxin Luo, Yun Xiang, Lizhe An

**Affiliations:** MOE Key Laboratory of Cell Activities and Stress Adaptations, School of Life Sciences, Lanzhou University, Lanzhou 730000, China; liujt@lzu.edu.cn (J.L.); zhouyuelong302@163.com (Y.Z.); 15125767698@163.com (C.L.); xiangy@lzu.edu.cn (Y.X.)

**Keywords:** salinity stress, *Achnatherum splendens*, time course, differentially expressed gene, expression patterns

## Abstract

*Achnatherum splendens* is an important forage herb in Northwestern China. It has a high tolerance to salinity and is, thus, considered one of the most important constructive plants in saline and alkaline areas of land in Northwest China. However, the mechanisms of salt stress tolerance in *A. splendens* remain unknown. Next-generation sequencing (NGS) technologies can be used for global gene expression profiling. In this study, we examined sequence and transcript abundance data for the root/leaf transcriptome of *A. splendens* obtained using an Illumina HiSeq 2500. Over 35 million clean reads were obtained from the leaf and root libraries. All of the RNA sequencing (RNA-seq) reads were assembled de novo into a total of 126,235 unigenes and 36,511 coding DNA sequences (CDS). We further identified 1663 differentially-expressed genes (DEGs) between the salt stress treatment and control. Functional annotation of the DEGs by gene ontology (GO), using *Arabidopsis* and rice as references, revealed enrichment of salt stress-related GO categories, including “oxidation reduction”, “transcription factor activity”, and “ion channel transporter”. Thus, this global transcriptome analysis of *A. splendens* has provided an important genetic resource for the study of salt tolerance in this halophyte. The identified sequences and their putative functional data will facilitate future investigations of the tolerance of *Achnatherum* species to various types of abiotic stress.

## 1. Introduction

Soil salinization is a serious problem that limits agricultural productivity and plant distribution and is detrimental to the ecological environment [1,2]. Results of a 2005 United Nations Food and Agriculture Organization (FAO) survey indicated that approximately 8 × 10^8^ hm^2^ of cultivated land were threatened by salinization [3], and the salinized area appears to be increasing at an alarming rate [4,5,6]. Therefore, better understanding of plant salt tolerance mechanisms is crucial for sustainable agricultural development and even for maintenance of environmental balance [7].

Salinity stress causes osmotic stress, oxidative stress, and ion toxicity in plant cells, resulting in cell dehydration, turgor pressure reduction, biochemical process disruption, and photosystem impairment [8,9,10]. To survive under salt stress conditions, plant cells rapidly sense the salt stress and respond with a series of complex alterations as follows: (1) changes in signaling and regulatory pathways [6,11,12], which are generally regulated by plant hormones [13,14]; (2) activation of specific transcription factors involved in the salinity stress response [15]; and (3) frequent regulation of the expression of genes that encode proteins required for osmoregulation, cell protection and/or acclimation [3,16,17,18]. These responses in plant cells may result in changes in many pathways, including signal transduction, ion homeostasis, reactive oxygen species (ROS)-scavenging, and growth regulatory pathways [6,19,20,21]. However, different plant species may have evolved various adaptive salt tolerance mechanisms. Therefore, comprehensive investigations of the transcriptomes of major halophytes are necessary to better understand the genetic mechanisms of salt tolerance.

*Achnatherum splendens* Trin. (Achnatherum) is an important forage herb that is widely distributed in the semi-arid northwestern region of China. This plant is considered to be one of the most important constructive plants in saline and alkaline areas given its high tolerance to salt, drought, and cold [22]. It is also an ideal model herb for the study of plant adaptation to these abiotic stresses [22,23,24]. *A. splendens* has evolved several morphological characteristics, including tough, slender leaves and well-developed root systems, to adapt to drought, salinization, and other environmental conditions. Therefore, this plant is also used as an excellent forage and textile plant to aid in soil and water conservation [25] and sewage treatment [26]. However, previous studies have exclusively focused on the ecological function and biochemistry of this species [22,26,27], and knowledge of its salt tolerance mechanisms are limited because genomic data on *A. splendens* are unavailable. *De novo* analysis using next-generation sequencing (NGS) technologies provides a robust platform for elucidating the genetic mechanisms of *A. splendens* salinity and alkalinity tolerance.

Here, we report the transcriptome of the entire *A. splendens* seedling under continuous salt stress. We assembled the transcriptome *de novo* and annotated the genes that were differentially expressed in response to salt stress. The results indicated that salinity-dependent metabolic pathways were activated that may be involved in ion transport, transcription, cellular communication and metabolism. These findings suggest that gene expression is highly coordinated in *A. splendens* in response to salinity stress. Furthermore, the identified DEGs provide an important genetic resource for further analyses of plant tolerance to salinity and molecular design breeding for the development of salt-tolerant forage herbs.

## 2. Materials and Methods

### 2.1. Plant Materials and Growth Conditions

Seeds of *Achnatherum splendens* Trin. (Achnatherum) were collected in Northwestern China. The *A. splendens* seeds were then sterilized with a 4% sodium hypochlorite solution for 15 min and rinsed thoroughly with sterile water. Next, filter paper was spread over the bottom of Petri dishes (12 cm in diameter and 3 cm in height, with a 13 cm-diameter lid, sealed with Parafilm), and the seeds were placed into the dishes with 20 mL MS (Murashige and Skoog Basal Medium with Vitamins, *Phyto* Technology Laboratories, Usage: 4.43 grams per liter) fluid nutrient medium containing different salt concentrations (0, 200, 300, 400, 500, and 600 mM NaCl, respectively). After culturing for 10 days, the chlorophyll concentrations in leaves were measured according to the protocol of Koski [28]. Similarly, leaf electrolyte leakage (EL) was measured according to Ishitani *et al.* [29]. Each treatment was replicated five times. Approximately fifty seeds per dish were placed at 22 °C for three days to measure the rate of seed germination. The seedlings were initially grown under identical non-stressed conditions in Petri dishes with MS nutrient solution for 15 days. Then, the control seedlings were further grown in the same nutrient solution, while the treated seedlings were transferred into new Petri dishes with MS nutrient solution containing 300 mM NaCl. All seedlings were grown in a greenhouse at 22 °C and with a 16/8 light/dark cycle (100 µmol·m^−2^·s^−1^). The morphological characteristics of fifteen-day-old seedlings exposed to the control and 300 mM NaCl treatments for 6 and 24 h were examined, and total RNA was extracted. For each treatment, ten seedlings from one dish were pooled and used as a single sample, and samples collected from three dishes were used as three biological replicates, respectively.

### 2.2. Total RNA Extraction and Sequencing

Total RNA for transcriptome sequencing was extracted using the cetyltrimethylammonium bromide (CTAB) procedure [30]. RNA was extracted from a total of nine samples (with the leaves and roots), including controls and samples exposed to 300 mM NaCl for 6 and 24 h, with three biological replicates each. The quality and concentration of the RNA samples were examined using a Nano Drop 1000 Spectrophotometers (Thermo Scientific Inc., Wilmington, DC, USA). A total of nine cDNA libraries were constructed and sequenced by Annoroad Technologies (Beijing, China). Standardized procedures were used and were monitored with a standard Quality Control System. RNAs with poly(A) tails were purified from total RNA using oligonucleotide (dT) magnetic beads and fragmented into short sequences that were used as templates for cDNA synthesis. After end repair, adapter ligation and PCR amplification, paired-end cDNA libraries were constructed and sequenced using an Illumina HiSeq 2500 sequencing platform (Illumina Inc., San Diego, CA, USA), with a 125-bp read length. Image output data from the sequencer were transformed into raw sequence data by base calling. These data were stored in FastQ format software [31].

### 2.3. Raw Sequence Processing and De Novo Assembly

To obtain high-quality reads, all raw reads were initially processed, including clean reads with adaptors removed. Reads with more than 5% ambiguous bases or “Ns” and those that contained over 20% Q < 20 bases were discarded [32]. The resulting clean reads were assembled using Trinity *de novo* assembly software [33], which constructs and reconstructs large regions of transcripts. First, contigs were generated by combining overlapping reads of a certain length. Only one read was retained per each assembly event; redundant duplicated reads were removed. These paired-end reads were then mapped to contigs. The paired-end reads align with contigs of the same transcript, and the intervals among them are measured. Finally, redundant sequences (with 95% similarity) of the high-quality unigenes were eliminated by CD-Hit [34]. All of the transcripts were further assembled into a single set of unigenes [35]. Next, *A. splendens* coding DNA sequences (CDSs) were identified and annotated using the “TransDecoder” module of the Trinity package with the default settings. Transcripts with reads per kilobase of transcript per million mapped reads (RPKM) values of below one in all libraries were deleted to ensure that all of the included transcripts were detectable.

### 2.4. Gene Expression Data

To measure the gene expression levels, Bowtie2 [36] was first used to align the RNA-seq reads to *A. splendens* transcriptome. Transcript abundances were determined by estimating read counts and RPKM values. Average RPKM values were calculated from the three biological replicates. The RPKM values of genes related to the salt response were log_2_-transformed and used to generate a heat map with the pheatmap package of R software.

### 2.5. Identification and Functional Analysis of DEGs

We measured the fold changes in expression of the genes between the salt-stressed and control *A. splendens* seedling samples to identify the DEGs and then identified the homologous genes. The DEGs were identified using the Empirical Analysis of Digital Gene Expression in R (edgeR) ver. 2.6.12 statistical package [37]. In pairwise comparisons performed with edgeR, the estimateGLMCommonDisp, and exactTest settings were used. The *P*-values obtained were adjusted to account for the false discovery rate (FDR) using the *p*.adjust function. An absolute fold change of >2 and an FDR of <0.001 were used as thresholds to identify significant differences in gene expression. The DEGs were first annotated using the top *Arabidopsis* and rice hits. Subsequently, gene ontology (GO) enrichment analysis was performed according to the three main GO ontologies: molecular function, cellular component, and biological process.

### 2.6. Validation of Expression of Genes Related to Trichome Initiation by Quantitative Real-Time PCR (qRT-PCR)

To validate the reliability of the RNA-Seq results, the expression of six candidate genes in response to salt stress were measured by qRT-PCR. A total of 0.5 μg total RNA treated with DNase I was converted into single-stranded cDNA using a Prime-Script First-Strand cDNA Synthesis Kit (TaKaRa, Dalian, China). The cDNA templates were then diluted 20-fold prior to use. qRT-PCR was performed with a CFX96 RealTime PCR Detection System (Bio-Rad, Singapore, Singapore) using SYBR Premix ExTaq™ (TaKaRa, Dalian, China) with the following program: 30 s at 95 °C, followed by 40 cycles of 95 °C for 15 s and 60 °C for 30 s, and a final step at 72 °C for 20 s. All of the specific primers used were designed with PRIMER5 software (PREMIER Biosoft, Palo Alto, CA, USA) and are listed in Supplementary Table S1. The expression of each gene was analyzed in three biological replicates. Relative expression levels were normalized by expression of the internal reference gene elongation factor 1-alpha (*EF1A*) and were calculated using the 2^−∆∆Ct^ method [38].

## 3. Results and Discussion

**Figure 1:**
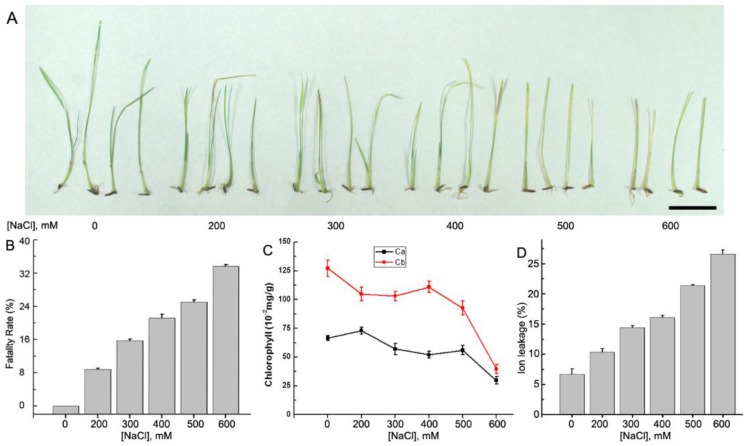
Salt responses of *A. splendens*. (**A**) Plant seedlings were treated with different concentrations of salt (0, 200, 300, 400, 500, and 600 mM NaCl for 10 days, respectively). Bar = 1 cm; (**B**) fatality rates of *A. splendens* control and treated seed samples; (**C**) the chlorophyll a and b concentrations in the control and treated seedlings; and (**D**) ion leakage from the leaves of control and treated seedlings. Each treatment was repeated five times.

NaCl stress decreased the rate of seed germination and seedling growth of *A. splendens*. To evaluate the effects of different concentrations of salt on the growth of *A. splendens* seedlings, we investigated the seed lethality rate and the leaf chlorophyll concentrations and ion leakage. The results showed that in the presence of high salt concentrations (600 mM NaCl), 34% of the seeds did not germinate. Even at moderate salt concentrations (300 mM NaCl), the lethality rate was 16% (Figure 1A,B). The chlorophyll a and b concentrations in the stressed leaves decreased significantly under both moderate (200–500 mM NaCl) and severe (600 mM NaCl) salt stress (*p* < 0.01) (Figure 1C). Furthermore, ion leakage from the leaves consistently increased in the presence of increasing salt concentrations (Figure 1D). These results suggest that *A. splendens* seedlings are able to survive exposure to higher salt concentrations similar to other halophytes, such as desert poplar [39,40,41], *Mesembryanthemum crystallinum* [42], and *Thellungiella salsugine* [7].

### 3.1. RNA-Seq and De Novo Assembly of the A. Splendens Transcriptome

To characterize the transcriptomic responses of *A. splendens* to salinity stress, nine cDNA libraries were constructed for both salt-stressed leaves and roots (exposed to 300 mM NaCl for 6 and 24 h, respectively) and control leaves and roots. Each treatment included three biological replicates. In total, 354.4 million 125-bp paired-end clean reads (45 Gb of sequence data) were generated by Illumina sequencing after the trimming of adapter sequences and removal of low-quality sequences (Supplementary Table S1). All of the RNA-seq reads were assembled *de novo* into a total of 125,235 high-quality unigenes (Supplementary File S1). Overall, 36,511 CDSs were recovered for *A. splendens* (Supplementary File S2). The average length of the CDSs was 1084 bp, with an N50 of 1356 bp (Supplementary Table S2). The reads were mapped to predicted unigenes, and the expression levels of the genes were calculated according to the RPKM values.

The unigenes were annotated using GO to clarify their predicted functions. GO is a classification system that uses standardized terms for assigning functions to uncharacterized sequences. A total of 21,590 unigenes were assigned to at least one GO term and were further classified into three groups. We further annotated these CDSes based on the top hits from *Arabidopsis* and rice retrieved from the Blastp program, with a cut-off e-value of 1 × 10^−5^. A total of 31,808 (87.1%) and 33,475 (91.6%) predicted genes were annotated using *Arabidopsis* and rice, respectively, suggesting that the sequence assembly was accurate.

### 3.2. Differential Gene Expression and Functional Analysis

**Figure 2:**
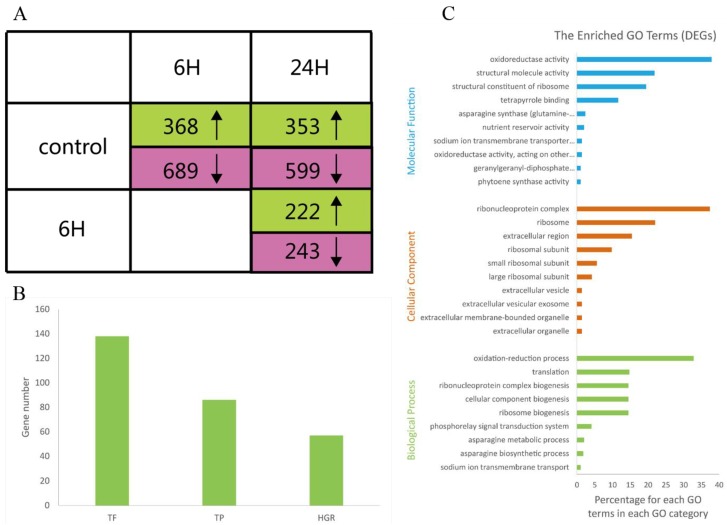
Transcriptomes analysis of *A. splendens* with three comparisons: (i) 6 *vs.* 0 h; (ii) 24 *vs.* 0 h; and (iii) 24 *vs.* 6 h. (**A**) Numbers of DEGs that were upregulated (green) and downregulated (pink) in the three comparisons (FDR <0.001; upregulated: fold change >2; and downregulated: fold change <0.5); (**B**) numbers of total DEGs and DEGs annotated as transcription factors (TFs), transporter proteins (TPs), and hormone-related genes (HRGs); and (**C**) GO terms that were overrepresented among the DEGs (color figure online).

**Figure 3:**
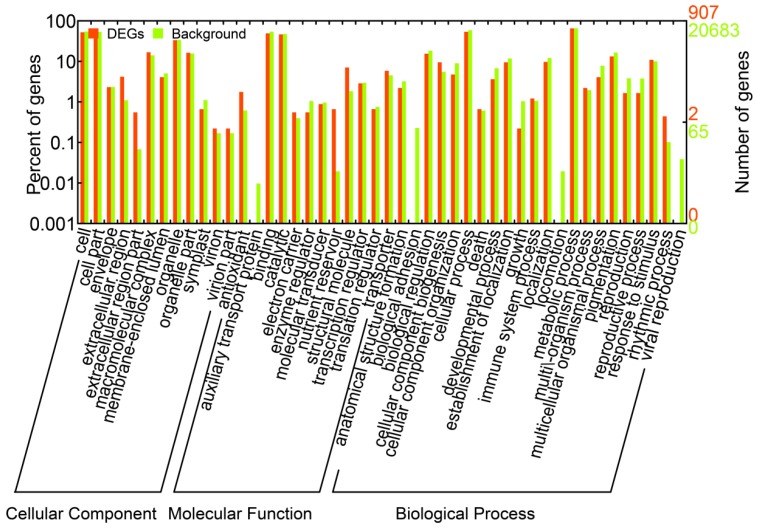
Gene ontology classifications of all of the unigenes and DEGs in *A. splendens*.

We identified a total of 1,663 DEGs that were upregulated or downregulated between the samples (6 *vs.* 0 h, 24 *vs.* 0 h, and 24 *vs.* 6 h), with fold changes of >2 or <0.5 and FDRs <0.001 (Supplementary Table S3). The numbers of DEGs (upregulated, downregulated, and total) are listed in Figure 2A. Most of the DEGs were downregulated at 6 h and upregulated at 24 h. These results suggested that during the salt treatments, the expression patterns of these genes differed at these two time points; thus, the *A. splendens* seedlings may have experienced salt shock (6 h) and then salt stress (24 h). Salt shock caused by exposure to a high concentration of salt may result in cell plasmolysis, leading to strong and rapid changes in the expression of genes with osmotic functions and slight and earlier changes in the expression of ion response genes. In contrast, more long-term salt stress causes relatively steady changes in the expression of genes related to osmotic stress (the first phase of salt stress) and dramatic changes in the expression of genes involved in the ionic phase of salt stress [43]. To elucidate the functions of the DEGs, an overview of the results was obtained using Web Gene Ontology Annotation Plot (WEGO), and the DEGs were assigned to GO categories grouped in the three main ontologies (Figure 3). The highest numbers of genes were annotated as having “metabolic” or “binding” functions compared with the other categories.

To further elucidate the functions of the DEGs, GO enrichment analysis was performed using Fisher’s exact test, with an adjusted FDR of *p* < 0.001 as the cutoff. A total of 29 GO terms were found to be enriched, as shown in Figure 2C. Most of these GO terms were associated with salt tolerance. For example, the description of the GO term “GO:0015081” is sodium ion transmembrane transporter activity. These results support previous findings that genes related to sodium ion transmembrane transporters are induced by salinity stress and are involved in salt tolerance in plants [7,39,42,44]. Other enriched GO terms were related to nutrient reservoirs and were assigned to the genes m.74667, m.2497, and m.32114, which were downregulated, indicating that nutrient levels were reduced during salt stress. Oxidoreductase activity also contributed to the salt tolerance of *A. splendens*. Similar results have been reported in the response of *A. splendens* to Pb^2+^ stress [5].

### 3.3. Functional Categories of DEGs Determined by Annotations to Arabidopsis Sequences

The DEGs were further classified as genes that encoded transcription factors, transporter proteins and hormones under salt stress and were further analyzed based on annotations to *Arabidopsis* sequences (Supplementary Tables S4–S6). Genes encoding transcription factors were of particular interest considering their abilities to control the expression of numerous genes and, thereby, regulate biological pathways and salt-related processes. Numerous transcription factors, including Mybs, AP2/ERF, bHLH, and bZIPs, are key regulators of gene expression and have been demonstrated to be induced in model plant species (such as *Arabidopsis thaliana* and rice) in response to abiotic stress [21]. Among the 1663 DEGs, 138 were identified as transcription factors in *A. splendens* and were further classified into 38 gene families with gene numbers of >2 (listed in Table 1).

A total of 86 DEGs in salt-stressed *A. splendens* were categorized as transporters (Supplementary Table S5). For example, gene m.39376, a homolog of sodium/hydrogen exchanger 2 (AT3G05030, CPA), m.138034, a homolog of Ca^2+^-transporting ATPase (AT2G41560), and m.54854, a homolog of chloride channel protein CLC-a (AT5G40890, CIC), were found to be involved in the salt tolerance of *A. splendens*. Taken together, these results and previous findings suggest that genes related to ion transport and chloride channels are strongly induced in response to salinity stress. Thus, these genes likely function to maintain or re-establish ion homeostasis in the cytoplasm of plant cells [19,39,41,45,46].

In addition, numerous hormone-related genes were induced or suppressed during the salt treatments [13,14,47]. Interestingly, approximately one-third of these genes were ABA-related genes (Supplementary Table S6). Based on previous findings, ABA is a hormone that is involved in regulation of the salinity response [48]. Thus, these results indicate that ABA is one of the main hormones responsible for the salt stress response of *A. splendens*.

### 3.4. Identification of Salt Tolerance Genes Using Rice Homologs

**Figure 4:**
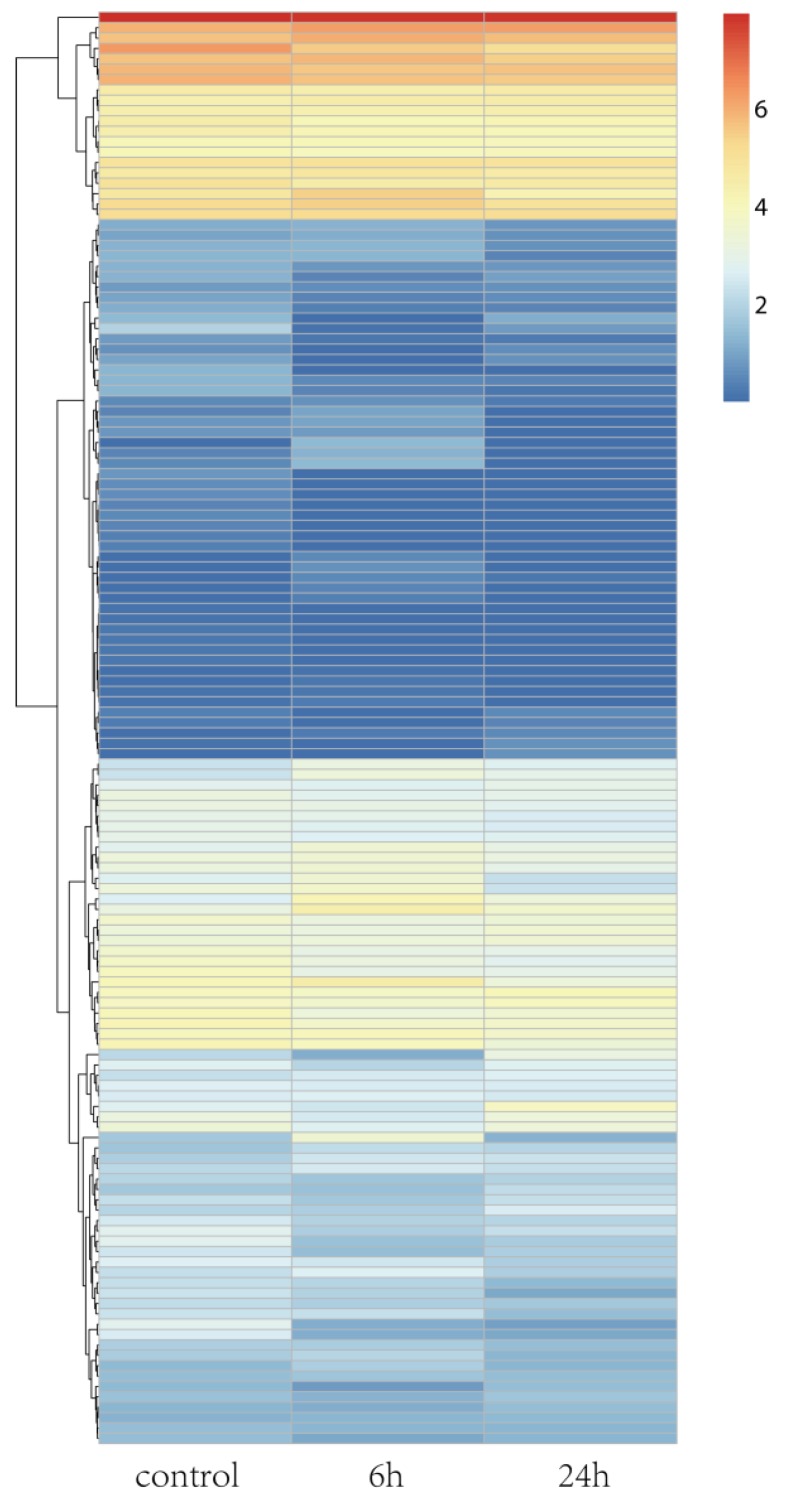
Hierarchical cluster analysis of DEGs under salt treatment in *A. splendens*.

Rice is a model plant, and its salt tolerance mechanisms have been well studied. *A. splendens* and rice both belong to the *Gramineae* family. Thus, we determined the salt tolerance genes in rice. Then, we identified their homologs in *A. splendens* and further examined their expression in response to salt tolerance. A total of 138 homologs were identified, and a heat map was generated for visualization of their expression patterns (Figure 4). The expression levels of most of the homologs were altered in response to salt stress. Among them, 18 were classified as DEGs (Figure 4 and Table 2). These genes encoded a “vacuolar Na^+^/H^+^ antiporter” (OsNHX1), “late embryogenesis abundant protein” (OsLEA3-2), and “potassium transporter” (OsHAK1). These results indicate that ion transport is crucial to the survival of *A. splendens* in high-salt environments. Thus, protein-coding genes involved in the maintenance of ion homeostasis in the cytoplasm may be strongly induced by salinity stress [19,39,41].

### 3.5. Validation of DEGs by qRT-PCR

**Figure 5:**
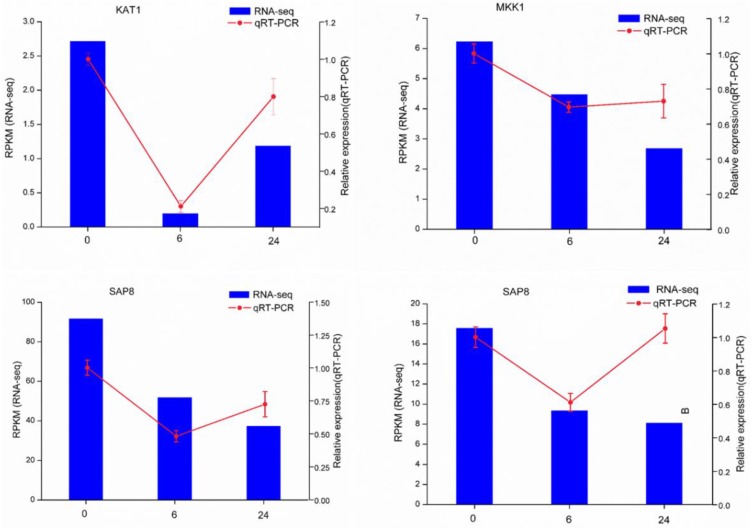
qRT-PCR verification of four selected DEGs. Comparison of RNA-seq data (blue bar) with qRT-PCR data (red line). The normalized RNA-seq expression levels (RPKM) are indicated on the y-axis to the left. The relative qRT-PCR expression levels are indicated on the y-axis to the right. The *EF1A* gene was used as an internal control. The results of both methods are consistent, with similar gene expression patterns (color figure online).

To validate the Illumina-Solexa sequencing results, four candidate DEGs associated with the salt response were selected for expression analysis by qRT-PCR, including one encoding mitogen-activated protein kinase kinase (MAPKK1), one encoding transporter protein (KAT1), and two encoding stress-associated protein (SAP8) (Supplementary Table S7). Although the fold changes in their expression detected by sequencing and qRT-PCR did not match exactly, the expression patterns determined for all four genes were consistent, confirming the reliability of the RNA-seq results (Figure 5).

### 3.6. The Proposed Mechanism of the A. Splendens Response to Salt Stress

**Figure 6:**
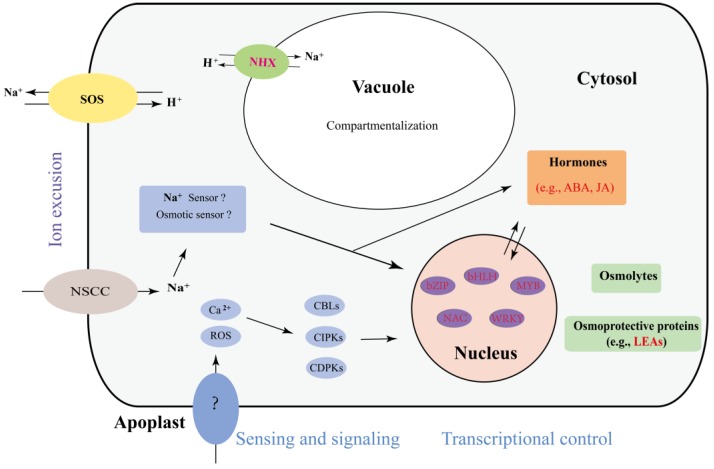
The predicted model of the *A. splendens* salt stress response. The genes presented in red are DEGs.

The mechanisms of plant salt stress responses are complex and have been well studied. To compare the mechanism used by *A. splendens* with those used by other plants, we summarized the findings of previous reports and generated a diagram of the salt response mechanism, as shown in Figure 6. This diagram presents the following three main mechanisms of plant tolerance to salt stress. (i) Plants increase their resistance to salt by exporting ions. Genes such as salt overly-sensitive (SOS) play important roles in this mechanism, and we found that their expression was altered in this study (Supplementary Table S4). (ii) The vacuole is an essential organelle that helps plants to transport ions to aid in their protection from salt stress [49]. Previous reports have indicated that NHX is involved in this process [44,49]. The differential expression of several NHX genes was identified in this study; thus, NHX proteins may play a role in the salt tolerance of *A. splendens*. (iii) Exposure of plants to salt stress results in alterations in the expression of genes encoding transcription factors and hormones to promote the increased production of osmolytes and osmoprotective proteins [50]. LEA is one of these proteins, and it has been demonstrated to be a main gene involved in the salt stress response [50]. Interestingly, we found several differentially-expressed LEA genes in *A. splendens,* indicating that it plays an important role in the salt stress response mechanism in this organism. In summary, we found that *A. splendens* responds to salt stress mainly via the transport of ions and osmotic adjustment.

## 4. Conclusions

In the present study, we explored the transcriptomic changes of *A. splendens* seedlings in response to salt stress over time and identified numerous DEGs with primary involvement in ion transport, transcription, cellular communication, and metabolism. Furthermore, we found that the temporal expression patterns of the DEGs varied greatly according to the length of time that the seedlings were exposed to the stress. We also found that the most of the DEGs were downregulated at 6 h of salt exposure but were upregulated at 24 h of salt exposure. These findings suggest that gene expression in *A. splendens* is highly coordinated in response to salinity shock at 6 h and salinity stress at 24 h. Furthermore, the identified DEGs provide an important genetic resource for future studies of plant tolerance to salinity and molecular design breeding for the development of salt-tolerant forage herbs.

## Figures and Tables

**Table 1:** Transcription factors differentially expressed in *A. splendens*.

Transcription Factor Family	Number of Genes
bHLH	15
bZIP	11
C2C2-Dof	3
DBB	5
GARP-G2-like	14
HB	3
HSF	4
MYB	4
MYB-like	7
NAC	8
NF-YC	6
Nin-like	4
OFP	3
Pseudo-ARR-B	10
TRAF	3
Trihelix	3
WRKY	5

**Table 2:** DEGs involved in the salinity response and their homologs in the rice genome.

Gene ID	Control	6 h	24 h	*FDR*	Rice Homolog	Function
m.155459	10.5	7.9	21.26	8.07 × 10^−8^	OsHsfC1b	heat shock transcription factor
m.184302	9.53	12.6	5.12	7.74 × 10^−8^	OsNHX1	vacuolar Na^+^/H^+^ antiporter
m.37867	0.11	2.34	0.25	8.81 × 10^−5^	OsLEA3-2	late embryogenesis abundant protein
m.39376	1.29	2.34	0.94	1.37 × 10^−5^	OsNHX1	vacuolar Na^+^/H^+^ antiporter
m.40521	6.24	18.4	4.11	1.54 × 10^−9^	Oshox22	homeobox-leucine zipper protein HOX22
m.43934	0.16	4.2	0.92	0.00086	OsLEA3-2	late embryogenesis abundant protein
m.44033	5.49	2.87	11.06	5.52 × 10^−14^	DCA1	Transcriptional co-activator of DST; CHY zinc finger protein
m.83348	6.35	9.8	4.25	4.10 × 10^−8^	OsNHX1	vacuolar Na^+^/H^+^ antiporter
m.135953	17.7	9.44	8.2	4.98 × 10^−6^	OsiSAP8	*O. sativa* subspecies *indica* stress-associated protein
m.47323	6.26	4.5	2.71	0.00051	OsMKK1	mitogen-activated protein kinase kinase
m.54442	7.06	2.26	1.95	5.50 × 10^−14^	OsHAK1	potassium transporter
m.54443	6.08	2.32	2.18	3.53 × 10^−11^	OsHAK1	potassium transporter
m.68120	1.64	0.1	0.01	2.64 × 10^−13^	OsCYP2	peptidyl-prolyl cis/trans isomerase; cyclophilin 2
m.76341	92.1	52.3	37.69	4.99 × 10^−12^	OsiSAP8	*O. sativa* subspecies *indica* stress-associated protein
m.159577	21.6	54.4	29.45	4.17 × 10^−5^	Oshox22	Homeobox-leucine zipper protein HOX22
m.6654	0.27	2.23	0.23	0.00027	OsLEA3-2	late embryogenesis abundant protein
m.68132	2.73	0.21	1.2	0.00011	OsKAT1	potassium channel
m.83942	9.49	22.9	13.8	1.66 × 10^−17^	OsGMST1	Golgi-localized monosaccharide transporter

#*FDR*: false discovery rate; the *FDR* threshold is 0.001.

## Supplementary Materials

The following tables are available online at www.mdpi.com/2073-4425/7/4/12/s1. File S1: Sequences of unigenes. File S2: Sequences of CDSs. Table S1: Output statistics of Illumina-Solexa sequencing; Table S2: Summary of the *A. splendens* transcriptome assembly; Table S3: Differentially expressed genes; Table S4: Transcription factor-encoding DEGs; Table S5: Transporter protein-encoding DEGs; Table S6: Hormone-encoding DEGs; and Table S7: Primers used for real-time quantitative RT-PCR in this study.
