# Supplementary material for: De Novo Transcriptome Sequencing of Desert Herbaceous Achnatherum splendens (Achnatherum) Seedlings and Identification of Salt Tolerance Genes
Source: Genes (Basel). 2016 Mar 23;7(4):12. doi: 10.3390/genes7040012 (PMC4846842; doi:10.3390/genes7040012)
Supplement: Supplementary file 1 [file genes-07-00012-s001.zip › genes-07-00012-supplementary/Table S1.docx]

**Table S1.** Output Statistics of the Illumina-Solexa sequencing.

| Sample ID |  | Total Reads | Reads length | Total Nucleotides (nt) * |
| --- | --- | --- | --- | --- |
| Control | 1 | 39267376 | 125 | 4908422000 |
|  | 2 | 38520328 | 125 | 4815041000 |
|  | 3 | 38711848 | 125 | 4838981000 |
| 6H | 1 | 38834522 | 125 | 4854315250 |
|  | 2 | 39213140 | 125 | 4901642500 |
|  | 3 | 40112302 | 125 | 5014037750 |
| 24 h | 1 | 39637694 | 125 | 4954711750 |
|  | 2 | 39873172 | 125 | 4984146500 |
|  |  | 40183072 | 125 | 5022884000 |
| Total |  | 354353454 |  | 44294181750 |
