# Supplementary material for: De Novo Transcriptome Sequencing of Desert Herbaceous Achnatherum splendens (Achnatherum) Seedlings and Identification of Salt Tolerance Genes
Source: Genes (Basel). 2016 Mar 23;7(4):12. doi: 10.3390/genes7040012 (PMC4846842; doi:10.3390/genes7040012)
Supplement: Supplementary file 1 [file genes-07-00012-s001.zip › genes-07-00012-supplementary/Table S2.docx]

**Table S2.** Summary of the transcriptome assembly for *A. splendens.*

| **Unigenes** |  |
| --- | --- |
| Number of unigenes | 126235 |
| Total length of unigenes (bp) | 153253758 |
| Average length of unigenes (bp) | 1214 |
| N50 of unigene length (bp) | 1692 |
| N90 of unigene length (bp) | 621 |
| Range of unigene length | 201–15993 |
| **CDS** |  |
| Number of CDS | 36511 |
| Total length of CDS (bp) | 39579468 |
| Average length of CDS (bp) | 1084 |
| N50 of CDS length (bp) | 1356 |
| N90 of CDS length (bp) | 537 |
| Range of CDS length | 201–14988 |
