# Supplementary material for: De Novo Transcriptome Sequencing of Desert Herbaceous Achnatherum splendens (Achnatherum) Seedlings and Identification of Salt Tolerance Genes
Source: Genes (Basel). 2016 Mar 23;7(4):12. doi: 10.3390/genes7040012 (PMC4846842; doi:10.3390/genes7040012)
Supplement: Supplementary file 1 [file genes-07-00012-s001.zip › genes-07-00012-supplementary/Table S7.docx]

**Table S7.** Primers used for real-time quantitative RT-PCR in this study.

| Gene | Annotation | Forward Primers(5’-3’) | Reverse Primers(5′-3′) |
| --- | --- | --- | --- |
| m.68132 | *KAT1* | TGTATCTGGAGCAGTGGA | AAAGTGAACGGCTGTGGT |
| m.47323 | *MKK1* | TGGGAAAGGTAGTAGTGGA | GGACAATCTGTTTGCGTAT |
| m.76341 | *SAP8* | TCAATAACTGTGGCTTCTTCG | CTCCTGCTTCATTATCATCTCC |
| m.135953 | *SAP8* | CACTTGTAGAAAGAGGGTCG | CATGCTTGTCAGAATAGCG |
| EF1A |  | ACCACGAGTCTCTTCTTGAGGCAC | TGGCAGGGTCATCCTTGGAG |
